# *Notes from the Field:* Live Poultry Shipment Box Sampling at Feed Stores as an Indicator for Human *Salmonella* Infections — Michigan, 2016–2018

**DOI:** 10.15585/mmwr.mm6817a6

**Published:** 2019-05-03

**Authors:** Jennifer L. Sidge, Kimberly Signs, Sally Bidol, Kelly Jones, Sheri Robeson, Marty Soehnlen, Mary Grace Stobierski

**Affiliations:** ^1^Bureau of Epidemiology and Population Health, Michigan Department of Health and Human Services; ^2^Bureau of Laboratories, Michigan Department of Health and Human Services.

*Salmonella* infection is estimated to cause 1.2 million human illnesses, 23,000 hospitalizations, and 450 deaths in the United States each year (*1*). An estimated 11% of *Salmonella* illnesses annually are caused by animal contact (*2*); contact with live poultry has become an increasing public health concern as backyard flock ownership has grown in popularity (*3*). Backyard poultry are usually purchased at agricultural feed stores that source birds from mail-order hatcheries (*4*). When a hatchery doesn’t have enough poultry to fill an order, it will often enlist a second hatchery as a source. Using a common industry practice, the second hatchery will drop-ship (i.e., ship under the original hatchery’s name and address) the poultry directly to the mail-order customer (retail feed store or individual customer) (*5*).

During human *Salmonella* outbreak investigations, real-time environmental sampling at mail-order hatcheries rarely occurs because of challenges involved in tracing poultry to its source. Environmental swabbing of arriving shipments and shipping information has been used to characterize *Salmonella* strains (*6*). This report describes an efficient method for detecting outbreak strains in live poultry by sampling poultry shipment box bedding/liners upon arrival at agricultural retail feed stores.

During 2016–2018, the Michigan Department of Health and Human Services (MDHHS) sampled live poultry shipping boxes at agricultural feed stores in Michigan. Upon arrival at stores, the bedding/liners from poultry boxes were placed into sterile collection bags by health department personnel with gloved hands. Photos were taken of the postal service labels to document shipment origin. In the case of drop-shipping, although the address of the hatchery to which the order was placed appears on the shipping label, postal service labels indicate the origin of the shipment. Samples were cultured, screened by polymerase chain reaction, and characterized by conventional serotyping and molecular subtyping processes (pulsed-field gel electrophoresis and whole genome sequencing of isolates) by MDHHS Bureau of Laboratories.

During 2016–2018, a total of 136 samples were collected at three agricultural feed store chains in 20 different Michigan locations, primarily during the spring months. The sampled boxes originated in nine different hatcheries, with approximately 65% originating at a single hatchery in Michigan. Thirty-five samples (26%) were culture-confirmed as six different serotypes of *Salmonella enterica*; of these, molecular subtyping linked four subtypes (Enteritidis, Braenderup, Muenster, and Senftenberg) with human illness outbreaks that occurred during 2016–2018. Results were shared with local health officials and the sampled agricultural feed store.

Sampling of poultry shipment boxes upon arrival at agricultural feed stores in the spring can provide an early indicator of *Salmonella* species present in hatchery-sourced live poultry. For example, in 2018, shipping box sampling occurred during February–March, and the first human cases in Michigan in which any live poultry exposure was reported had illness onsets in April, at least a month later. Sampling poultry shipment boxes is also quick, easy, and can have high yields.

The mail-order hatchery industry practice of drop-shipping (*5*) can complicate traceback investigations because hatchery records must be requested and reviewed to discover the actual source of poultry. Using postal service labels to determine hatchery of origin is an efficient method for tracking the source to a specific hatchery and simplifies traceback during outbreak investigations. Implicated hatcheries can be notified about infections earlier and mitigation steps initiated sooner, potentially resulting in fewer human illnesses. Testing for the presence of *Salmonella*, either by sampling shipment boxes or sampling at the implicated hatchery directly, provides hatcheries with early vital information to inform appropriate *Salmonella* mitigation strategies.
