# *Erratum:* Vol. 68, No. 17

**DOI:** 10.15585/mmwr.mm6830a5

**Published:** 2019-08-02

In the report “Notes from the Field: Live Poultry Shipment Box Sampling at Feed Stores as an Indicator for Human *Salmonella* Infections — Michigan, 2016–2018,” on page 407, the sentence **“These findings corroborate previously published results that found a positive correlation between the *Salmonella* molecular strains found in shipment boxes and those serotypes associated with human illnesses (*7*).**” should have been included at the end of the fourth paragraph. In addition, on page 408, the reference “**7. Sharma A, Erdman MM, Munoz-Vargas L, Mollenkopf DF, Habing GG. Changes in the prevalence, genotypes and antimicrobial resistance phenotypes of non-typhoidal *Salmonella* recovered from mail-order hatchling poultry sold at US feed stores, 2013–2015. Zoonoses Public Health 2018;65:e102–12**,” should have been included in the list of references.

